# High-Intensity Intermittent Training Positively Affects Aerobic and Anaerobic Performance in Judo Athletes Independently of Exercise Mode

**DOI:** 10.3389/fphys.2016.00268

**Published:** 2016-06-28

**Authors:** Emerson Franchini, Ursula F. Julio, Valéria L. G. Panissa, Fábio S. Lira, José Gerosa-Neto, Braulio H. M. Branco

**Affiliations:** ^1^Department of Sport, School of Physical Education and Sport, University of São PauloSão Paulo, Brazil; ^2^Exercise and Immunometabolism Research Group, Department of Physical Education, Paulista State UniversityPresidente Prudente, Brazil

**Keywords:** combat sports, high-intensity intermittent training, blood lactate, muscle damage markers, oxygen uptake, hormones

## Abstract

**Purpose:** The present study investigated the effects of high-intensity intermittent training (HIIT) on lower- and upper-body graded exercise and high-intensity intermittent exercise (HIIE, four Wingate bouts) performance, and on physiological and muscle damage markers responses in judo athletes.

**Methods:** Thirty-five subjects were randomly allocated to a control group (*n* = 8) or to one of the following HIIT groups (*n* = 9 for each) and tested pre- and post-four weeks (2 training d·wk^−1^): (1) lower-body cycle-ergometer; (2) upper-body cycle-ergometer; (3) *uchi-komi* (judo technique entrance). All HIIT were constituted by two blocks of 10 sets of 20 s of all out effort interspersed by 10 s set intervals and 5-min between blocks.

**Results:** For the upper-body group there was an increase in maximal aerobic power in graded upper-body exercise test (12.3%). The lower-body group increased power at onset blood lactate in graded upper-body exercise test (22.1%). The *uchi-komi* group increased peak power in upper- (16.7%) and lower-body (8.5%), while the lower-body group increased lower-body mean power (14.2%) during the HIIE. There was a decrease in the delta blood lactate for the *uchi-komi* training group and in the third and fourth bouts for the upper-body training group. Training induced testosterone-cortisol ratio increased in the lower-body HIIE for the lower-body (14.9%) and *uchi-komi* (61.4%) training groups.

**Conclusion:** Thus, short-duration low-volume HIIT added to regular judo training was able to increase upper-body aerobic power, lower- and upper-body HIIE performance.

## Introduction

Judo athletes' training is directed to technical, tactical, or conditioning improvements via complementary (non-specific) and specific actions (Franchini et al., 2014). Traditionally, a typical judo microcycle involves aerobic, strength, and judo skills training (Miura et al., 2005; Mochida et al., 2007; Yamamoto et al., 2008; Franchini and Takito, 2014).

Since 2009, with the new International Ranking List system, introduced by the International Judo Federation, international level judo athletes take part in 5–10 competitions per year, to obtain points to qualify for the Olympic Games (Franchini and Takito, 2014). As the competitions are distributed throughout the year, the athletes need to interperse high-intensity, recovery, and tapering training periods to achieve their best performance in the competitions. The period of intensified training has as the main goal improving physical conditioning by focusing on aspects relevant to competitive success (Franchini and Takito, 2014; Franchini et al., 2014). Considering that competitive judo is intermittent in nature (Miarka et al., 2012, 2014; Franchini and Takito, 2014) and presents high physiological demand (Franchini et al., 2011, 2014), high-intensity intermittent training (HIIT) is highly used in judo training sessions (Franchini et al., 2014), especially via technique entrance (*uchi-komi*) (Baudry and Roux, 2009; Franchini et al., 2013b; Franchini and Takito, 2014). Thus, considering the number of competitions per year, the recovery needed after a competition and the tapering phase before the next one, it is common that the intensified training be conducted over short (typically 4-week) periods.

Using non-athletes as subjects, several studies reported that HIIT programs were able to improve aerobic and anaerobic performance and increase physiological indexes related to them (Linossier et al., 1997; MacDougall et al., 1998; Burgomaster et al., 2005; Gibala et al., 2006). The positive and expressive results observed via these training protocols, associated with the short time period needed to conduct these sessions, have attracted researchers and trainers due to its excellent cost-benefit approach (Buchheit and Laursen, 2013). Consequently, studies were conducted with athletes from cyclical sports (Laursen et al., 2005), with positive results being observed and recommendations being proposed to increase athletes' performance (Laursen, 2010; Buchheit and Laursen, 2013).

Studies with combat sports, such as karate (Ravier et al., 2009) and wrestling (Farzad et al., 2011) also used HIIT protocols. Ravier et al. (2009) submitted nine karate athletes to an additional training protocol constituted of seven to nine 20 s running bouts at 140% $\overset{\cdot }{V}$O_2max_, with 15 s intervals, twice per week, over 7 weeks, and found increased time to exhaustion (23.6%), maximal accumulated oxygen deficit (10.3%), and $\overset{\cdot }{V}$O_2max_ in a graded treadmill exercise test (4.6%). Conversely, the group that maintained only the regular karate training did not improve in any of these variables. Farzad et al. (2011) submitted wrestlers to a short (4 week) HIIT program, with two sessions per week (six 35 m sprints, with 10 s intervals), and reported increased $\overset{\cdot }{V}$O_2max_ in the graded treadmill exercise test (5.4%), time limit at $\overset{\cdot }{V}$O_2max_ (32.2%), and higher peak (11.9% and 36.5%) and mean power (6.5% e 9.1%) in the first two of four Wingate tests. However, the training induced creatine quinase increase at rest (20.3%), indicated increased muscle damage during this training protocol. Additionally, an increased peak blood lactate after the Wingate tests was observed, as well as increased testosterone/cortisol ratio, suggesting also improved metabolic and hormonal profiles. However, no change was observed in the control group.

As judo is a sport demanding use of both lower and upper-body during the match, it is common that judo athletes be submitted to cycle-ergometer training protocols to develop these body regions (Franchini et al., 2014), although judo-specific exercises are also used, as the *uchi-komi* (Baudry and Roux, 2009; Franchini et al., 2013b). Thus, the objective of the present study was to verify the effects of three different HIIT protocols on aerobic and anaerobic performance and physiological responses compared to a control group submitted only to judo training. It was expected that judo athletes submitted to lower or upper-body exercise modes would present better adaptation to the specific mode trained (i.e., higher increase in aerobic and anaerobic performance in upper-body for the upper-body training group and higher increase in lower-body to the lower-body training group), while the *uchi-komi* group would present an intermediary improvement in both segments as this mode activates both lower- and upper-body segments and the control group would no present any significant increase in these parameters.

## Materials and methods

### Subjects

Thirty-five male judo athletes were recruited to take part in the present study. Signed consent was sought and obtained from athletes or from their guardians in case they were underage in the year this study was conducted. Groups did not differ concerning the age at the beginning of the study (*P* > 0.05): lower-body training group = 22.3 ± 5.2 years; upper-body training group = 23.6 ± 6.7 years; *uchi-komi* training group = 23.4 ± 4.2 years; control group = 26.4 ± 7.0 years.

Athletes were from eight different judo clubs and competed with each other at the state level. All procedures were approved by the Ethics and Research Committee of the School of Physical Education and Sport, University of São Paulo. To take part in this study the athletes were required to present the following characteristics: (I) to have taken part in official judo competitions during the current year; (II) to be training at least four times per week; (III) aged equal to or higher than 17 years old and < 35 years-old; (IV) competed in the under 100-kg categories; (V) not involved in any weight-loss programs; (VI) not involved in any supplementation or medical treatment. At the time of the experiment athletes were engaged in four 1.5–2 h judo sessions per week and in three strength training sessions per week. The time of judo practice for each training group was: lower-body (*n* = 9) = 12 ± 7 years; upper-body (*n* = 9) = 15 ± 7 years; *uchi-komi* (*n* = 9) = 12 ± 7 years; control group (*n* = 8) = 18 ± 7 years. Groups did not differ concerning time of judo practice (*P* > 0.05).

### Study design

This study adopted a completely randomized experimental design. Three groups of judo athletes were randomly formed and submitted to 4-week HIIT programs directed to lower-body, upper-body or throwing technique entrance (*uchi-komi*) added to the regular judo training, while a control group only performed the regular judo training group. After familiarization with all test procedures, athletes were submitted to a physical fitness test battery, before and after 4 weeks of training, consisting of: (a) an upper-body cycle-ergometer graded exercise test, where peak oxygen uptake ($\overset{\cdot }{V}$O_2peak_), maximal aerobic power (MAP), maximum heart rate (HR_max_), and power (P_OBLA_) and oxygen uptake ($\overset{\cdot }{V}$O_2OBLA_) at onset blood lactate accumulation (OBLA) were determined; (b) a lower-body cyclergometer graded exercise test, where $\overset{\cdot }{V}$O_2peak_, MAP, HR_max_, P_OBLA_, and $\overset{\cdot }{V}$O_2OBLA_ were determined; (c) four upper-body Wingate tests with 3-min intervals, where peak (PP) and mean power (MP) and total work (TW) performed were determined; (d) four lower-body Wingate tests with 3-min intervals, where PP, MP, and TW performed were determined and muscle damage markers and blood lactate responses measured at rest and after the tests; (e) anthropometrical measurements, including body mass, height, and body fat percentage. The test battery was conducted on two non-consecutive days, 1 week before the training protocol started and 3 days after the ending of the 4-week training.

Aerobic and intermittent tests were conducted for different body segments on each day, with 45-min interval between tests. The same test sequence was adopted in the post-test period, at the same time of day, to prevent performance and physiological variations associtated to the circadian rhytm.

### Anthropometry

The following anthropometric measurements were carried out: body mass, height, skinfold thicknesses (triceps, subscapular, supraspinale, abdominal, front thigh, and medial calf), bone diameters (biacromial, chest, chest depth, biiliac, humerus, and femur epicondyles) and circumferences (thorax, relaxed arm, wrist, proximal thigh, medial calf, and ankle). Skinfold thickness measurements (Harpenden plicometer; John Bull British Indicators, England; constant pressure of 10 g/mm and precision of 0.2 mm) were carried out three times at each point in a rotation system, as described by Drinkwater and Ross (1980). Tests were conducted by a researcher with more than 10 years of experience in this measurement procedure, presenting a variation of < 2.0% between measurements, with reproducibility determined by an intra-class correlation coefficient more than 0.98, within the assessment period. The circumferences and bone diameters were measured only once at each point by the same experienced evaluator, presenting < 0.9% variation between measurements. Body fat percentage was determined using the method proposed by Drinkwater and Ross (1980).

### Lower- and upper-body graded exercise tests

The participants performed two (one for upper-body and one for lower-body) incremental tests to volitional exhaustion in a cycle-ergometer to determine the $\overset{\cdot }{V}$O_2peak_, maximum heart rate HR_max_, MAP, $\overset{\cdot }{V}$O_2OBLA_, and P_OBLA_). For lower-body (Excalibur, Lode, Netherlands) the initial load was set at 70 W. Each stage lasted 3 min and was increased 35 W per stage until the athlete could no longer continue or maintain 70 rpm for more than 5 s (Denadai et al., 2004). For upper-body (EB 4100, Cefise, Brazil) the initial load was set at 26.5 W. Each stage lasted 3 min and was increased 26.5 W per stage until the athlete could no longer continue or maintain 90 rpm for more than 5 s (Pires et al., 2011). The oxygen uptake $\overset{\cdot }{V}$O_2_ (MetaMax^®^3B, *Cortex*, Germany) and heart rate (Polar *Electro Oy*, Finland) were measured throughout the test.

The maximal load reached in the test was defined as the maximal intensity attained. When the subject was not able to finish the 3-min stage, the power was expressed according to the permanence time in the last stage, determined as the following: power of penultimate stage + [(time, in seconds, remained at the last stage multiplied by increase)/180 s].

The $\overset{\cdot }{V}$O_2peak_ was established as the highest 15 s average value in the test. The HR_max_ corresponded to the maximum value obtained throughout the test.

At the end of each stage in both tests, there was an interval of 30 s to collect blood samples from the ear lobe to determine the lactate concentration (Yellow Spring 1500 Sport, Yellow Springs, United States) and to determine the power at OBLA (defined as the concentration equivalent to 3.5 mmol.L^−1^) (Heck et al., 1985). $\overset{\cdot }{V}$O_2_ at OBLA were also determined.

### Lower- and upper-body high-intensity intermittent performance tests

Judo athletes completed four bouts of Wingate tests separated by 3-min recovery periods one time for each body segment, i.e., lower- and upper-body. The load was set at 0.09 kg.kg^−1^ of body mass for lower-body test and 0.06 kg.kg^−1^ of body mass for upper-body test. Before Wingate tests a standard warm-up was conducted. It was composed of 5 bouts of 30-s (20-s at 70 rpm, and 10-s at 100 rpm), with ~100 W for lower-body and 50 W for upper-body. They started the Wingate test after 3-min of interval, and left from zero velocity. For the lower-body version an Excalibur (Lode, Netherlands) cycle-ergometer was used, while for the upper-body version the test was conducted using an EB 4100 cycle-ergometer (Cefise, Brazil). PP, MP, and TW were calculated as previously reported (Franchini et al., 2003). Blood samples from the ear lobe were taken to determine the lactate concentration (Yellow Spring 1500 Sport, Yellow Springs, United States) before each test, 1 and 2.5-min after the first three bouts and 1, 3, and 5-min after the fourth bout, and delta of lactate was calculated (peak lactate concentration after the test minus the lactate concentration at rest).

### Venous blood sampling and analyses

Venous blood samples were collected by venous puncture from an antecubital vein (15 mL) pre- and post-training period before and after the lower- and upper-body high-intensity intermittent tests. Athletes always performed the test on the same schedule (afternoon and evening). They reported an adequate hydration status and sleep before the day of each testing protocol. Blood samples were collected at rest in a lying position and room temperature (~20°C) on all days for all athletes to eliminate fluctuations in circulating analyte concentration because of circadian rhythm.

The blood samples (15 mL) were immediately allocated into two 5 mL vacutainer tubes (Becton Dickinson, BD, Juiz de Fora, MG, Brazil) containing EDTA for plasma separation and into one 5 mL dry vacutainer tube for serum separation. The tubes were centrifuged at 3500 g for 12 min at 4°C, and plasma and serum samples were stored at −80°C until analysis. To eliminate inter-assay variance, all samples were analyzed in identical runs, resulting in an intra-assay variance of < 5%. Testosterone and cortisol were assessed using ELISA commercial kits (Monobind Inc. 100 North Point Drive, Lake Forest, CA 92630 USA). Creatine kinase (CK), lactate dehydrogenase (LDH), aspartate aminotransferase (AST), alanine aminotransferase (ALT) were assessed using commercial kits (Labtest^®^, São Paulo, Brazil). The testosterone and cortisol levels were assessed using plasma, and CK, LDH, AST, and ALT levels were assessed using serum. As there is evidence (Ravier et al., 2009; Farzad et al., 2011) that no change would ocurr in the control group, the venous blood samples were not taken and, thus, the muscle damage marker and hormonal responses were not measured for this group.

### Training protocols

As in the studies of Ravier et al. (2009) with karate athletes and Farzad et al. (2011) with wrestlers, the groups were submitted to complementary HIIT twice a week. The lower- and upper-body groups performed a 5-min warm-up at 40% of MAP. For the lower-body group the warm-up was conducted at 70 rpm, while for the upper-body group cadence was set at 90 rpm, each on the specific ergometer. The warm-up for the *uchi-komi* group was also 5-min in duration, with movements and entrances of the techniques without throwing the other athlete, who had a similar body mass as the executant (difference set at ±10% of body mass). After the warm-up the athletes rested 3-min before starting the proposed protocol. The training session lasted for a total of 22 min 40 s, taking into account the 5-min warm-up, 3-min rest after warm-up, one block of 4 min 50 s of high-intensity intermittent exercise (ten times 20 s effort by 10 s pause), 5-min recovery and the 2nd block of high-intensity intermittent workout. The main difference in the groups undergoing training was the way the high-intensity intermittent exercise was conducted: (I) the lower-body group performed the HIIT using a lower-body cycle ergometer. These stimuli consisted of two blocks of 10 sets of 20 s with 10 s intervals between sets and 5-min between blocks. Each effort was an all-out bout using 4.5% of body mass as resistance; (II) the upper-body group underwent exactly the same protocol used by the lower-body group, except that the ergometer used was an upper-body bicycle and the load used was 3% of body mass; (III) the specific group was submitted to two blocks of 10 sets of 20 s of the *uchi-komi* (technique entrance), throwing the partner at the end of each set, with 10 s intervals between sets and a 5-min interval between blocks. Similar to the other groups, the athletes in this group were also asked to perform each set in an all-out mode. The technique entrance was conducted on the tatami, and athletes were asked to perform either *te-waza* (arm techniques, such as *morote-seoi-nage, ippon seoi-nage*) or *koshi-waza* (hip techniques, such as *o-goshi, harai-goshi*, and *koshi-guruma*). During the execution of *uchi-komi* athletes were also requested to execute the pulling action and the technique execution of its complete form and to throw their partner at the end of each 20 s period.

The choice of this temporal structure was due to the following factors: (I) judo fights have an average duration of 3-min, with actions performed in about 10 sequences lasting around 20 s, with 10 s interval between them (Miarka et al., 2012); (II) these protocols can be executed in < 15 min, including period-of-rest intervals between sets and between blocks of exercise; (III) in each session, the total exercise time does not exceed 7-min, resulting in a small addition in the total weekly training volume.

The mean training load for the eight HIIT sessions, assessed via the method proposed by Foster et al. (2001), did not differ (*P* > 0.05) between the experimental groups: lower-body training group = 171.7 ± 51.5 a.u.; upper-body training group = 168.6 ± 55.8 a.u.; *uchi-komi* training group = 190.8 ± 45.0 a.u.

### Statistics

All analyses were performed using the Statistica software (version 12). Data were reported as means and standard deviation (SD). A Mauchly's test of sphericity was used to test this assumption and as it was confirmed there was no need to use the Greenhouse-Geisser correction. A two-way (group and period of training) analysis of variance with repeated measures was applied to compare groups and period of training for anthropometrical measurements, performance in the variables derived from the graded exercise tests and total work during lower- and upper-body high-intensity intermittent tests. A three-way (group, period of training and test bout or moment of measurement) analysis of variance with repeated measurements was used to compare blood parameters before and after each lower- and upper-body Wingate bout, MP and PP during each lower- and upper-body Wingate bout. Bonferroni's *post-hoc* test correcting for multiple comparisons was used when a significant interaction between two or three factors was found in the ANOVA. When only the period of training factor was found, paired Student *t*-tests was conducted for each group to verify the effect of each training protocol. For ANOVA results, effect sizes were calculated using partial eta squared (η^2^), which were classified according to Cohen (1969) using the following scale for interpretation: < 0.2 [small]; 0.2 to < 0.8 [moderate]; >0.8 [large]; while for *t*-tests results, effect sizes were calculated using Cohen'd as proposed by Rhea (2004) using the following scale (highly trained individuals) for interpretation: < 0.25 [trivial]; 0.25 to < 0.5 [small]; 0.5 to < 1.00 [moderate]; >1.0 [large]. Statistical significance was set at *P* < 0.05. Due to the large amount of data, statistics details for blood lactate in results (tables and figures) were presented only for significant results involving an effect of period of training or interaction between another factor and period of training or group (i.e., results concerning effect of moment of measurement are omitted in the text, except when interacting with period of training or group).

## Results

### Body mass and body fat percentage

There were no effects (*P* > 0.05) of group, training period or interaction for body mass (lower-body training group: pre = 76.9 ± 10.9 kg; post = 77.1 ± 10.7 kg; upper-body training group: pre = 84.2 ± 13.5 kg; post = 84.2 ± 12.6 kg; *uchi-komi* training group: pre = 78.1 ± 13.4 kg; post = 78.2 ± 12.5 kg; control group: pre = 80.2 ± 10.3 kg; post = 80.6 ± 11.2 kg) and body fat percentage (lower-body training group: pre = 12.8 ± 3.7%; post = 12.0 ± 3.0%; upper-body training group: pre = 15.5 ± 4.3%; post = 15.6 ± 4.1%; *uchi-komi* training group: pre = 15.4 ± 5.0%; post = 15.3 ± 3.6%; control group: pre = 14.6 ± 3.5%; post = 14.2 ± 4.2%).

### Upper- and lower-body aerobic tests

Table 1 presents the performance and physiological responses to the upper-body and lower-body maximal graded test for each group.

**Table 1:** **Performance and physiological responses to the upper-body and lower-body maximal cycle-ergometer graded test conducted by judo athletes pre and post different high-intensity intermittent training protocols (values are mean ± standard deviation)**.

	**Lower-body training group (*****n*** = **9)**		**Upper-body training group (*****n*** = **9)*****^#^***		***Uchi-komi*** **training group (*****n*** = **9)**		**Control group (*****n*** = **8)**	
	**Pre**	**Post**	**Pre**	**Post**	**Pre**	**Post**	**Pre**	**Post**
**UPPER-BODY MAXIMAL GRADED TEST**								
MAP (W)	136 ± 15	143 ± 14	146 ± 18^*^	164 ± 15	139 ± 25	149 ± 17	120 ± 20	117 ± 28
$\dot{V}$O_2peak_(L.min^−1^)	2.78 ± 0.41	3.03 ± 0.39	3.10 ± 0.70	3.22 ± 0.58	3.16 ± 0.30	3.27 ± 0.33	2.86 ± 0.37	2.72 ± 0.83
HRmax (bpm)	180 ± 11	183 ± 18	179 ± 11	179 ± 20	180 ± 7	178 ± 12	169 ± 9	169 ± 15
Power at OBLA (W)	68 ± 22^*^	83 ± 14	82 ± 21	95 ± 27	66 ± 15	72 ± 12	74 ± 26	71 ± 21
$\dot{V}$O_2_ at OBLA (L.min^−1^)	1.80 ± 0.35	2.14 ± 0.53	1.94 ± 0.40	2.00 ± 0.51	1.79 ± 0.32	1.75 ± 0.28	2.06 ± 0.35	2.06 ± 0.45
**LOWER-BODY MAXIMAL GRADED TEST**								
MAP (W)	240 ± 28	251 ± 49	259 ± 43	254 ± 39	252 ± 37	255 ± 36	217 ± 34	217 ± 35
$\dot{V}$O_2peak_(L.min^−1^)	3.62 ± 0.50	3.68 ± 0.80	3.82 ± 0.59	3.86 ± 0.44	3.87 ± 0.44	3.74 ± 0.36	3.56 ± 0.49	3.54 ± 0.74
HRmax (bpm)	179 ± 8	180 ± 13	183 ± 9	185 ± 18	181 ± 12	178 ± 10	174 ± 9	169 ± 23
Power at OBLA (W)	150 ± 15	149 ± 38	160 ± 35	163 ± 24	136 ± 31	148 ± 17	142 ± 22	138 ± 23
$\dot{V}$O_2_ at OBLA (L.min^−1^)	2.24 ± 0.24	2.19 ± 0.52	2.23 ± 0.56	2.47 ± 0.37	2.10 ± 0.29	2.12 ± 0.27	2.53 ± 0.33	0.46

MAP, Maximal aerobic power; $\overset{\cdot }{V}$O_2_ peak, peak oxygen consumption; HRmax, maximal heart rate; OBLA, onset blood lactate accumulation;

* different from post-training in the same group (P < 0.05).

# Different from control group (P < 0.05).

No significant (*P* > 0.05) differences were found for $\overset{\cdot }{V}$O_2peak_, HRmax, and $\overset{\cdot }{V}$O_2OBLA_ for the upper-body aerobic test. For MAP, an effect of group [*F*_(3, 30)_ = 4.90; *P* = 0.007; η^2^ = 0.328; moderate] was detected, with higher values in upper-body group compared to the control group (*p* = 0.005). There was also an effect of period of training [*F*_(1, 30)_ = 6.88; *P* = 0.013; η^2^ = 0.186; small], with higher values post- compared to pre-training (*P* = 0.013), which was a consequence of the higher post- values compared to pre-training for the upper-body group [*t*_(7)_ = −3.653; *P* = 0.008; *d* = 1.04; large].

For P_OBLA_, an effect of period of training [*F*_(1, 29)_ = 5.61; *P* = 0.024; η^2^ = 0.162; small] was observed, with higher values post- compared to pre-training (*p* = 0.024), and the *t*-test demonstrated that only for the lower-body group values were higher post- compared to pre-training [*t*_(8)_ = −2.894; *P* = 0.020; *d* = 0.69; moderate].

For the lower-body aerobic test no significant (*P* > 0.05) differences were found for any variable (MAP, and $\overset{\cdot }{V}$O_2peak_, HRmax, P_OBLA_, and $\overset{\cdot }{V}$O_2OBLA_).

### Upper- and lower-body high-intensity intermittent test

**Figure 1:**
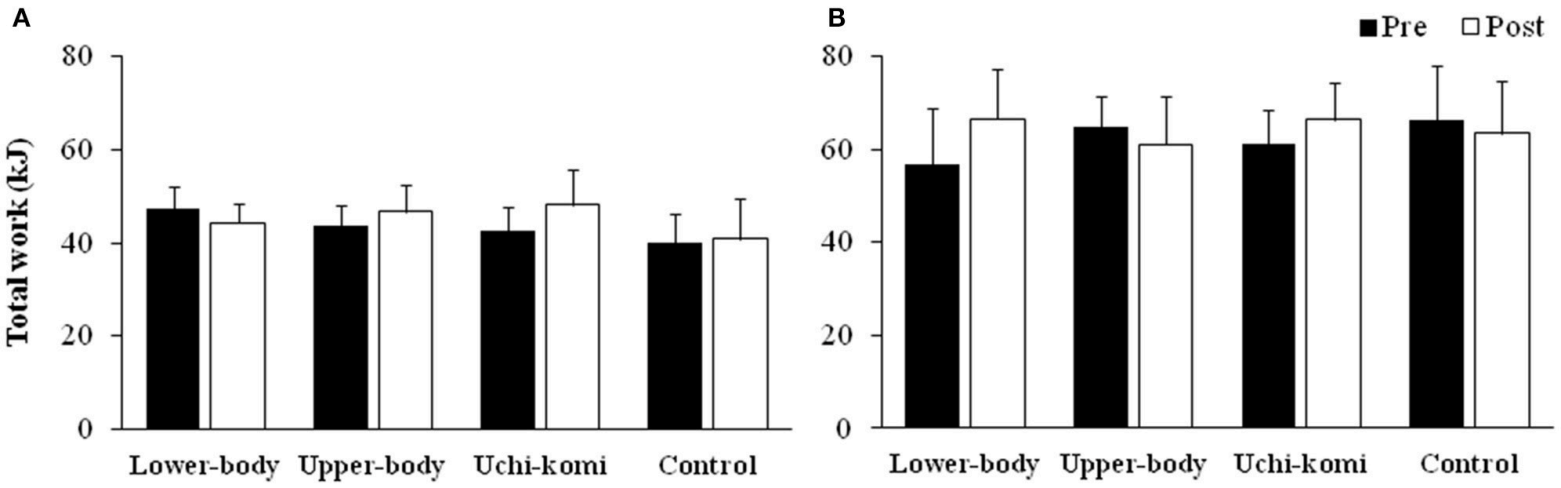
**Total work (kJ) during four bouts of upper- (A) and lower-body (B) Wingate tests pre- and post-training in judo athletes submitted to different high-intensity intermittent training protocols**.

Figure 1 presents TW during four bouts of upper- and lower-body Wingate tests pre- and post-training in judo athletes in the control group and those submitted to different HIIT protocols.

There was a period of training and group interaction effect for upper-body TW [*F*_(3, 29)_ = 4.72; *P* = 0.008; η^2^ = 0.328; moderate] and for lower-body TW [*F*_(3, 27)_ = 4.56; *P* = 0.010; η^2^ = 0.336; moderate], but the *post-hoc* test did not confirm any difference (*P* > 0.05).

### Upper-body high-intensity intermittent test performance and physiological responses

**Figure 2:**
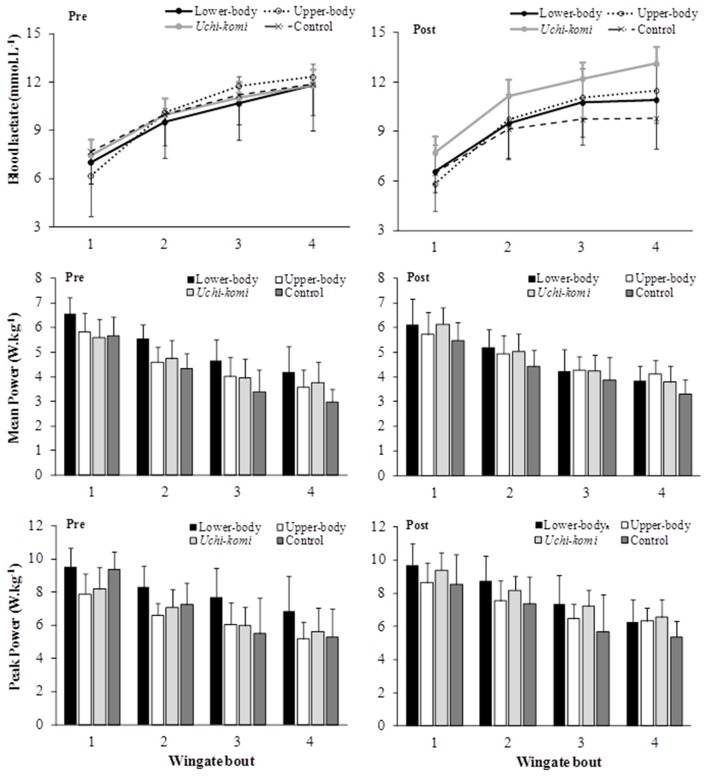
**Blood lactate, mean, and peak power responses to four bouts of upper-body Wingate tests in judo athletes submitted to different high-intensity intermittent training protocols**. ^*^Pre-training different from post in the same group (*P* < 0.05).

Figure 2 presents delta blood lactate, PP and MP during four bouts of upper-body Wingate tests pre- and post-training in judo athletes in the control group and those submitted to different HIIT protocols.

For upper-body delta blood lactate no significant (*P* > 0.05) differences were found. For upper-body MP, there was period of training and group interaction effect [*F*_(3, 29)_ = 4.68; *P* = 0.009; η^2^ = 0.327; moderate], although the *post-hoc* did not confirm any difference. For upper-body PP, there was a period of training and group interaction [*F*_(3, 29)_ = 4.91; *P* = 0.007; η^2^ = 0.337; moderate], with *uchi-komi* group showing higher values post- than pre-training (*P* = 0.015).

Table 2 presents the biochemical and hormonal responses to the upper-body Wingate bouts before and after training periods in the different groups.

**Table 2:** **Biochemical and hormonal responses to four upper-body Wingate bouts conducted by judo athletes pre and post different high-intensity intermittent training protocols (values are mean ± standard deviation)**.

		**Lower-body training group (*****n*** = **9)**		**Upper-body training group (*****n*** = **9)**		***Uchi-komi*** **training group (*****n*** = **9)**	
		**Pre**	**Post**	**Pre**	**Post**	**Pre**	**Post**
CK (U.L^−1^)	Before Wingate bouts^*^	492.9 ± 414.5	221.6 ± 240.0	348.0 ± 286.0	207.2 ± 106.3	498.5 ± 535.8	385.3 ± 458.6
	After Wingate bouts	665.5 ± 546.0	314.0 ± 304.4	460.6 ± 340.2	285.1 ± 123.4	700.5 ± 808.6	523.0 ± 559.0
LDH (U.L^−1^)^&^^§^^α^	Before Wingate bouts^*^	85.1 ± 65.6	78.4 ± 30.1	75.3 ± 35.9	75.6 ± 25.6	79.2 ± 19.3	79.0 ± 36.6
	After Wingate bouts	115.7 ± 80.1	92.0 ± 42.2	121.6 ± 94.0	109.7 ± 44.6	122.0 ± 69.6	101.9 ± 20.3
AST (U.L^−1^)	Before Wingate bouts^*^	5.3 ± 1.3	5.1 ± 1.7	4.6 ± 2.2	5.5 ± 2.5	4.8 ± 1.8	4.3 ± 1.4
	After Wingate bouts	5.7 ± 1.4	6.7 ± 3.0	6.3 ± 2.7	6.4 ± 2.6	6.1 ± 2.6	7.0 ± 3.9
ALT (U.L^−1^)	Before Wingate bouts^*^	3.13 ± 1.08^†^	3.38 ± 0.81	4.46 ± 2.69^†^	4.67 ± 2.41^†^	3.75 ± 1.63	3.57 ± 1.07^£^
	After Wingate bouts	4.46 ± 1.39	4.43 ± 1.21	6.58 ± 3.74	5.75 ± 3.11	4.68 ± 1.87	5.78 ± 1.75^#^
Cortisol (ng.mL^−1^)^‡^	Before Wingate bouts^*^	8.66 ± 4.35	11.62 ± 6.23	5.71 ± 1.95	7.56 ± 5.32	7.36 ± 3.98	5.60 ± 2.41
	After Wingate bouts	14.72 ± 5.66	17.15 ± 6.70	9.95 ± 5.72	9.36 ± 6.53	12.96 ± 5.13	11.42 ± 5.76
Testosterone (ng.mL^−1^)	Before Wingate bouts^*^	4.20 ± 1.86	4.63 ± 1.30	4.56 ± 1.65	5.10 ± 0.14	5.04 ± 0.21	5.06 ± 0.13
	After Wingate bouts	4.71 ± 1.46	4.70 ± 1.33	4.65 ± 1.65	5.20 ± 0.12	5.17 ± 0.17	5.15 ± 0.15
Testosterone/Cortisol ratio	Before Wingate bouts^*^	0.60 ± 0.33	0.58 ± 0.42	0.80 ± 0.34	0.95 ± 0.58	0.83 ± 0.34	1.05 ± 0.38
	After Wingate bouts	0.42 ± 0.33	0.35 ± 0.26	0.57 ± 0.35	0.82 ± 0.59	0.47 ± 0.22	0.63 ± 0.43

CK, creatine quinase; LDH, lactate dehydrogenase; AST, aspartato transaminase; ALT, alanine transaminase;

* = moment of measurement main effect, different from after Wingate bouts (P < 0.05);

† group, moment of measurement and training period interaction, different from the same group after the Wingate bouts at pre and post-training (P < 0.05);

£ group, moment of measurement and training period interaction, different from the same group before the Wingate bouts at pre-training (P < 0.05);

# group, moment of measurement and training period interaction, different from the same group at pre-training before and after Wingate bouts and at pos-training before Wingate bouts;

‡ group main effect, lower-body training group different from upper-body training group (P < 0.05);

& moment of measurement and training period interaction (P < 0.05), after at pre-training different from after at post-training;

§ moment of measurement and training period interaction (P < 0.05), after at pre-training different from before and after at post-training;

α moment of measurement and training period interaction (P < 0.05), before different from after at post-training.

For CK concentration response to the four upper-body Wingate bouts an effect of moment of measurement was also observed [*F*_(1, 21)_ = 35.08; *P* < 0.001; η^2^ = 0.626; moderate], with higher values post- compared to pre-Wingate bouts (*P* < 0.001).

For the LDH concentration response to the Wingate bouts an effect of moment of measurement was found [*F*_(1, 24)_ = 19.52; *P* < 0.001; η^2^ = 0.449; moderate], with higher values after the Wingate bouts compared to before the test (*P* < 0.001). A tendency of training period and moment of measurement interaction was detected [*F*_(1, 24)_ = 4.12; *P* = 0.054; η^2^ = 0.147; small], with lower values before the Wingate bouts in the pre-training period compared to after the Wingate bouts in the pre-training (*P* < 0.001) and post training (*P* = 0.006) periods, higher values after the Wingate bouts in the pre-training period compared to before (*P* < 0.001) and after (*P* = 0.020) Wingate bouts in the post-training periods and lower values before the Wingate bouts in the post-training period compared to after the test in the same period (*P* = 0.002).

The AST concentration response to the four upper-body Wingate bouts was affected by the moment of measurement [*F*_(1, 24)_ = 19.98; *P* < 0.001; η^2^ = 0.454; moderate], with higher values post- compared to pre-Wingate bouts (*P* < 0.001).

For ALT concentration response to the upper-body Wingate bouts an effect of moment of measurement was detected [*F*_(1, 24)_ = 98.53; *P* < 0.001; η^2^ = 0.804, large], with higher values after the Wingate bouts compared to before the tests (*P* < 0.001). Additionally, a group, training period and moment of measurement interaction was found [*F*_(2, 24)_ = 9.24; *P* = 0.001; η^2^ = 0.435, moderate], with lower values for the lower-body training group before the tests in the pre-training period compared to after the Wingate bouts in the pre-training (*P* = 0.004), lower values for the upper-body training group before the tests in the pre-training period compared to after the Wingate bouts in the pre-training (*P* < 0.001), lower values for the upper-body training group before the tests in the post-training period compared after the Wingate bouts in the post-training (*P* = 0.042), higher values for the *uchi-komi* training group after the tests in the post-training period compared and after (*P* = 0.034) the tests in the pre-training periods.

Cortisol concentration response to the upper-body Wingate bouts was affected by group [*F*_(2, 23)_ = 3.98; *P* = 0.032; η^2^ = 0.257; moderate] and moment of measurement [*F*_(1, 23)_ = 27.27; *P* < 0.001; η^2^ = 0.542; moderate]. The lower-body training group presented higher cortisol concentration than the upper-body training group (*P* = 0.037) and post-Wingate bout values were higher than before the test (*P* < 0.001).

An effect of moment of measurement [*F*_(1, 23)_ = 5.25; *P* = 0.032; η^2^ = 0.186; small] was detected for testosterone, with higher values post- compared to pre-Wingate bouts (*P* = 0.029).

Testosterone-cortisol ratio response to the upper-body Wingate bouts was affected only by moment of measurement [*F*_(1, 23)_ = 23.69; *P* < 0.001; η^2^ = 0.507; moderate], with lower values before the Wingate bouts than after (*P* < 0.001).

### Lower-body high-intensity intermittent test performance and physiological responses

**Figure 3:**
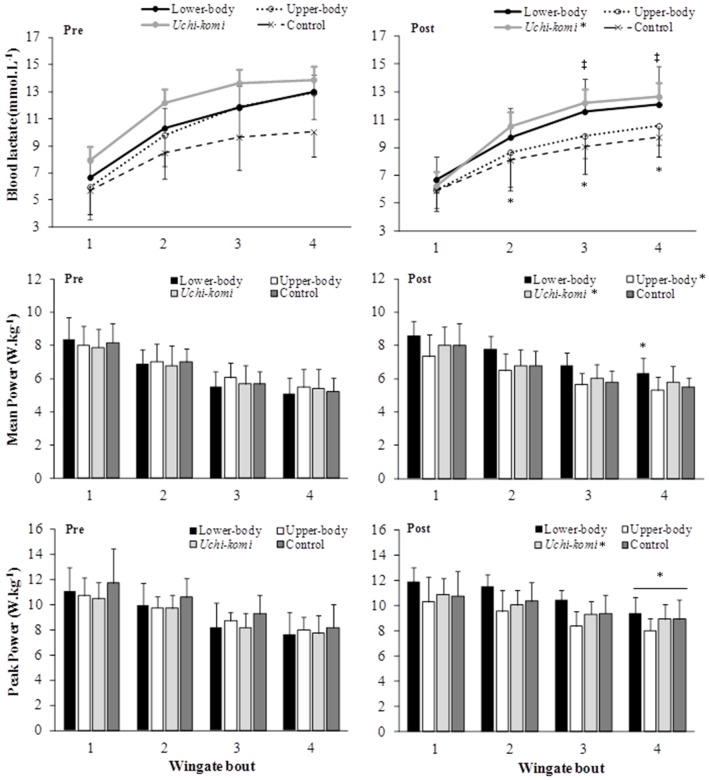
**Blood lactate, mean and peak power responses to four bouts of lower-body Wingate tests in judo athletes submitted to different high-intensity intermittent training protocols**. ^‡^ = Different from pre-training in upper-body group (*P* < 0.05); *pre- different from post-training in the same group training (*P* < 0.05).

Figure 3 presents delta blood lactate, PP and MP during four bouts of lower-body Wingate tests in judo athletes in the control group and those submitted to different HIIT protocols.

For lower-body delta blood lactate, there was a period of training effect [*F*_(1, 30)_ = 14.88; *P* < 0.001; η^2^ = 0.331; moderate], with higher pre- values than post-training (*P* < 0.001). A period of training and bout interaction effect [*F*_(3, 90)_ = 3.84; *P* = 0.012; η^2^ = 0.114; small] was observed, with higher pre- values compared to post- in bouts 2, 3, and 4 (*P* < 0.001 for all). For isolated groups a period of training and bout interaction effect to upper-body training group [*F*_(3, 24)_ = 4.31; *P* = 0.014; η^2^ = 0.350; moderate] was detected, with higher values in pre- compared to post-training in bouts 3 and 4 (*P* = 0.007; *P* = 0.011; respectivelly). Additionally, a period of training effect for *uchi-komi* training group [*F*_(1, 8)_ = 6.59; *P* = 0.033; η^2^ = 0.452; moderate] was observed, with higher values in pre- compared to post-training (*P* = 0.033).

For lower-body PP, there was a period of training effect [*F*_(1, 30)_ = 5.30; *P* = 0.028; η^2^ = 0.150; small], with higher values post- than pre-training (*p* = 0.030). There was a period of training and bout interaction effect [*F*_(3, 90)_ = 5.19; *P* = 0.002; η^2^ = 0.148; small], with lower values in pre- than post-training in bout 4 (*P* < 0.001). A period of training effect for the *uchi-komi* group was also observed [*F*_(1, 8)_ = 14.62; *P* = 0.005; η^2^ = 0.636; moderate], with lower values pre- than post-training (*P* = 0.005).

For lower-body MP, a period of training and group interaction effect [*F*_(3, 29)_ = 5.18; *P* = 0.005; η^2^ = 0.349; moderate], but the *post-hoc* test did not confirm any difference. A period of training and bout interaction [*F*_(3, 87)_ = 9.24; *P* < 0.001; η^2^ = 0.242; moderate] was detected, with higher values in bout 4 post- than pre-training (*P* = 0.002). A period of training and bout interaction effect in the lower-body training group [*F*_(3, 21)_ = 4.01; *P* = 0.021; η^2^ = 0.364; moderate] was found, with lower values in pre- than post-training in bout 4 (*P* = 0.007). A period of training effect to upper-body group [*F*_(1, 8)_ = 5.53; *P* = 0.047; η^2^ = 0.409; moderate] was observed, with higher values pre- than post-training (*P* = 0.047). Furthermore, a period of training for the *uchi-komi* group [*F*_(1, 8)_ = 7.04; *P* = 0.029; η^2^ = 0.468; moderate] was also found, with higher values pre- than post-training (*P* = 0.029).

Table 3 presents the biochemical and hormonal responses to the lower-body Wingate bouts before and after training periods in the different groups.

**Table 3:** **Biochemical and hormonal responses to four lower-body Wingate bouts conducted by judo athletes pre and post different high-intensity intermittent training protocols (values are mean ± standard deviation)**.

		**Lower-body training group (*****n*** = **9)**		**Upper-body training group (*****n*** = **9)**		***Uchi-komi*** **training group (*****n*** = **9)**	
		**Pre**	**Post**	**Pre**	**Post**	**Pre**	**Post**
CK (U.L^−1^)	Before Wingate bouts^*^	361.6 ± 379.3	335.2 ± 226.2	294.6 ± 250.5	184.4 ± 51.7	441.2 ± 615.8	394.4 ± 329.2
	After Wingate bouts	500.8 ± 409.8	464.4 ± 281.4	390.8 ± 313.0	224.9 ± 63.8	587.9 ± 849.9	542.5 ± 440.1
LDH (U.L^−1^)	Before Wingate bouts^*^	84.0 ± 51.6	68.8 ± 17.7	72.3 ± 15.9	75.6 ± 25.6	73.1 ± 18.2	90.9 ± 29.8
	After Wingate bouts	131.0 ± 101.9	119.6 ± 104.4	162.1 ± 169.9	109.7 ± 44.6	107.9 ± 22.6	126.8 ± 45.7
AST (U.L^−1^)	Before Wingate bouts^*^	4.5 ± 1.6	4.9 ± 1.0	4.8 ± 2.5	5.2 ± 2.5	5.1 ± 1.5	5.3 ± 2.3
	After Wingate bouts	5.0 ± 1.4	6.3 ± 1.8	6.6 ± 3.2	5.8 ± 2.7	6.4 ± 2.1	6.5 ± 2.1
ALT (U.L^−1^)	Before Wingate bouts^*^	2.92 ± 1.34	3.82 ± 2.08	4.35 ± 2.28	4.68 ± 1.78	3.54 ± 1.41	3.89 ± 1.66
	After Wingate bouts	3.94 ± 1.49	5.67 ± 1.91	6.14 ± 2.79	5.56 ± 2.55	5.85 ± 2.05	5.47 ± 1.81
Cortisol (ng.mL^−1^)^†^^‡^^&^	Before Wingate bouts^*^	12.13 ± 6.17^#^	11.70 ± 7.11	7.23 ± 4.43	7.64 ± 4.06	7.34 ± 4.46	5.43 ± 1.86
	After Wingate bouts	17.60 ± 7.11	13.31 ± 6.19	11.19 ± 5.28	12.17 ± 6.50	13.96 ± 5.30	8.15 ± 4.13
Testosterone (ng.mL^−1^)	Before Wingate bouts	4.65 ± 1.49	4.65 ± 1.20	4.58 ± 1.59	4.73 ± 1.13	5.10 ± 0.21	5.06 ± 0.09
	After Wingate bouts	4.70 ± 1.44	4.69 ± 1.09	4.65 ± 1.64	5.15 ± 0.15	5.17 ± 0.17	5.14 ± 0.16
Testosterone/Cortisol ratio^†^	Before Wingate bouts^*^	0.56 ± 0.41	0.61 ± 0.43	0.74 ± 0.41	0.88 ± 0.72	0.75 ± 0.45	1.03 ± 0.34
	After Wingate bouts	0.38 ± 0.34	0.47 ± 0.35	0.46 ± 0.24	0.66 ± 0.59	0.40 ± 0.24	0.81 ± 0.43

CK, creatine quinase; LDH, lactate dehydrogenase; AST, aspartato transaminase; ALT, alanine transaminase;

* = moment of measurement main effect, different from after Wingate bouts (P < 0.05);

† training effect, pre- different from post (P < 0.05);

# group and training period interaction, different from uchi-komi group at post-training (P < 0.05);

‡ moment of measurement and training period interaction, before at pre and post-training different from after at pre and post-training;

& moment of measurement and training period interaction (P < 0.05), after at pre-training different from after at post-training.

For CK concentration response to the four lower-body Wingate bouts an effect of moment of measurement was observed [*F*_(1, 19)_ = 37.65; *P* < 0.001; η^2^ = 0.665; moderate], with higher values post- compared to pre-Wingate bouts (*P* < 0.001).

For cortisol concentration response there were training period [*F*_(1, 24)_ = 7.00; *P* = 0.014; η^2^ = 0.226, moderate] and moment of measurement [*F*_(1, 24)_ = 22.95; *P* < 0.001; η^2^ = 0.489, moderate] effects. Higher values were observed pre- compared to post-training (*P* = 0.014) and higher values after the Wingate bouts compared to pre-Wingate bouts (*P* < 0.001). There were also group and training period [*F*_(2, 24)_ = 3.70; *P* = 0.040; η^2^ = 0.236, moderate] and training period and moment of measurement interactions [*F*_(1, 24)_ = 5.94; *P* = 0.023; η^2^ = 0.198, small]. There was a tendency for higher values for the *uchi-komi* group at pre- compared to itself at post-training (*P* = 0.058). Values before the Wingate bouts at pre-training were lower than after the Wingate bouts at pre-training (*P* < 0.001) and values before the Wingate bouts at post-training were lower than after the Wingate bouts at post-training (*P* = 0.002). Additionally, values after the Wingate bouts at pre- were higher than after the Wingate bouts at post-training (*P* = 0.001).

For testosterone concentration a tendency of moment of measurement effect [*F*_(1, 23)_ = 3.89; *P* = 0.061; η^2^ = 0.145, small] was detected, with a tendency to higher values post- compared to pre-Wingate bouts (*P* = 0.057).

Testosterone-cortisol ratio response to the lower-body Wingate bouts was affected by moment of measurement [*F*_(1, 24)_ = 30.97; *P* < 0.001; η^2^ = 0.563, moderate] and training period [*F*_(1, 24)_ = 5.25; *P* = 0.031; η^2^ = 0.179, small]. Higher values were observed before the Wingate bouts than after the Wingate bouts (*P* < 0.001) and lower values were measured at pre- compared to post-training (*P* = 0.031). Additionally, when each group was analized separately, lower values were observed pre- compared to post-training for the lower-body (14.9%; *P* = 0.035) and *uchi-komi* (61.4%; *P* = 0.026) training groups.

## Discussion

The main findings of the present study was that the addition of short-term (4 week) low-volume (twice per week) HIIT (two blocks of 10 sets of 20 s with 10 s intervals between sets and 5-min between blocks) to the traditional judo training resulted in increased (a) upper-body MAP for the upper-body training group, (b) upper-body P_OBLA_ for the lower-body training group, (c) PP in the upper-body intermittent test for the *uchi-komi* training group; (d) PP in the lower-body intermittent test for the *uchi-komi* training group; (e) MP in the fourth Wingate test in the lower-body intermittent test for the lower-body training group; (f) testosterone-cortisol ratio in the lower-body intermittent test for the lower-body and *uchi-komi* training groups. There was a decrease in the delta blood lactate for the *uchi-komi* training group and in the third and fourth bouts for the upper-body training group. No effects on any lower-body aerobic variable, muscle damage markers, body mass and body fat percentage were found. The only negative effects found were the decreased MP in the lower-body intermittent test for the upper-body and the *uchi-komi* training groups. As expected, no change was observed in the control group. Thus, the initial hypothesis that lower- and upper-body training group would adapt better to lower- and upper-body tests, respectively, and that the *uchi-komi* training group would present intermediary adaptation was partially confirmed.

Only upper-body aerobic performance was affected by the HIIT, and this improvement was observed specifically in the MAP of the upper-body training group. Improvement in aerobic power related variables are normally attributed to increases in oxygen delivery (i.e., elevated cardiac output) and to oxygen utilization by active muscles (i.e., arteriovenous difference) (Bassett and Howley, 2000), but in our study $\overset{\cdot }{V}$O_2peak_ did not increase as consequence of the training period. No study that analyzed MAP in upper-body aerobic test protocols in judo athletes was found. Specifically with combat sports, only two studies used supramaximal HIIT (Ravier et al., 2009; Farzad et al., 2011). Both studies used running as the exercise mode and observed similar $\overset{\cdot }{V}$O_2max_ magnitude increase: 5.4% for wrestlers submitted to 4 weeks of additional HIIT [20] and 4.6% in karate athletes submitted to 7 weeks HIIT program (Ravier et al., 2009). Thus, the results of the present study, with highly trained judo athletes, did not result in similar adaptations as previously reported in the literature. Additionally, for the upper-body aerobic test, increases in P_OBLA_ was observed only for the lower-body training group. No study analyzed anaerobic threshold-related variables in upper-body aerobic test protocols. For the lower-body aerobic test, the only study found using similar HIIT protocols and training duration measuring submaximal aerobic variables (i.e., ventilatory threshold 1 and 2) was one conducted by Laursen et al. (2005), reporting increases between 16 and 24% for the ventilatory threshold 2, improvements that are similar to those found in our study (22.1%). The effects of HIIT, using supramaximal bouts, on submaximal aerobic variables were not studied in combat sports athletes.

Judo athletes from the *uchi-komi* training group increased upper-body PP, suggesting that the interaction of judo training and the high-intensity *uchi-komi* additional training resulted in an optimal combination to improve upper-body high-intensity intermittent performance. Probably the lower stimulus directed to the upper-body for the lower-body training group and an excessive stimulus directed to the upper-body for the upper-body group did not interact with judo training to improve performance in this test. Complementary, as judo training does not result in elevated lower-body solicitation, the interaction between the typical judo training and the HIIT directed to the lower-body in the lower-body training group resulted in increased lower-body MP during the high-intensity intermittent test. Taken together, these results are probably due to the fact that HIIT anaerobic adaptations are mainly muscle-specific, as many key enzymes increase their activity after this kind of training protocol (MacDougall et al., 1998). Although the effects of HIIT on upper-body high-intensity intermittent protocols have not been studied, Farzad et al. (2011) reported an increase in PP and MP in the first two Wingate test bouts after 4 weeks of additional HIIT to regular wrestling training in the training group compared to the control group.

In our study lower-body PP and MP increased with training, and this improvement was observed in the fourth Wingate bout. Farzad et al. (2011) also reported improvement in lower-body PP and MP after a similar HIIT program, but their athletes increased performance in the first and second Wingate bouts (in a total of four bouts as used in our study). As a significant aerobic participation has been reported in the last bouts of this kind of protocol and we found a decreased blood lactate response for the *uchi-komi* training group and for test 3 and 4 for the upper-body training group, it is possible to infer that the muscle metabolic adaptation was different in our athletes compared to those in the study conducted by Farzad et al. (2011), because they did not detect any significant change in blood lactate response to the Wingate bouts in the trainining group. An increase in key aerobic enzymes has also been reported after HIIT training protocols (MacDougall et al., 1998) and it is possible that the increase in the last Wingate bouts performance and the decrease blood lactate in these bouts be a consequence of a higher muscle oxidative capacity. Additionally, the increased anabolic state, as evidenced by the increased testosterone-cortisol ratio in the lower-body intermittent test for the lower-body and *uchi-komi* training groups can also be an explanation for the improved high-intensity intermittent performance. Conversely, the decreased MP in the lower-body intermittent test specifically for the upper-body and the *uchi-komi* training groups can also be interpreted considering the judo-specific demand (Franchini et al., 2013a, 2014), i.e., as judo presents a high demand on upper-body, the small judo-specific lower-body power demand and the lack of specific stimulus for this body region in the upper-body and *uchi-komi* training groups did not result in improvements in PP.

The low volume short-term HIIT protocols, the moderate to low initial body fat percentage, the absence of nutritional intervention to reduce dietary consumption and the fact that judo athletes are categorized according to their body mass are factors that can explain the body mass and body fat percentage maintenance. Due to the low volume of HIIT protocols used in the present study the energy expenditure was probably small, as ten 60 s bouts interspersed by 60 s intervals were reported to result in 84 kJ (Skelly et al., 2014). Although studies reporting energy expenditure in an exactly-matched effort-pause duration cycle-ergometer high-intensity intermittent exercise protocol were not found in the literature, the total energy expenditure during such protocols has been considered small. However, for a 3-min high-intensity intermittent *uchi-komi* protocol (9 × 20 s all-out *harai-goshi* execution interpersed by 20 s intervals) the energy expenditure has been reported to be 314.2 ± 64.5 kJ (~34.9 kJ for each 20 s period) (Franchini et al., 2013b). Thus, the HIIT added to the regular judo training in the present study would result in only ~1396 kJ per week. In this sense a much longer period or a controlled energy intake promoting energy deficit would be needed to reduce body mass or body fat percentage in this group.

Basically, the muscle damage markers (CK, LDH, AST, and ALT) analyzed in the present study increased after both upper- and lower-body high-intensity intermittent protocol compared to pre-test values. Exercise intensity is one important aspect contributing to sarcolemma disruption, allowing the release of these enzymes into the blood (Brancaccio et al., 2007) and the high-intensity intermittent exercise protocols used in the present study are considered very demanding and have been applied to evaluate judo athletes' performance (Franchini et al., 2003, 2009). However, no effect of training period was observed. A HIIT study, using wrestlers as subjects, reported increased resting CK concentration after the 4-week training intervention (Farzad et al., 2011), but the athletes trained using running as an exercise mode, which could have induced a higher eccentric overload than the exercise modes used in the present study. In fact, the main factor contributing to muscle damage are the eccentric actions (Byrne et al., 2004), and the only group that practiced HIIT with eccentric actions was the *uchi-komi* training group, although the type of eccentric actions during technique repetitions are probably much less exacerbated than during running.

Both upper- and lower-body high-intensity intermittent protocols induced cortisol concentration elevation. For testetosterone the upper-body protocol resulted in an increase, while the lower-body protocol presented only a tendency to increase this hormone concentration. Testosterone-cortisol ratio also increased in response to both protocols. These results indicate the high hormonal disturbance caused by the high-intensity intermittent exercise.

However, more important to the present study's goals are the differences between training groups or periods. In this context, the cortisol response to the lower-body high-intensity intermittent exercise protocol was lower at pre-training compared to post-training, suggesting a less catabolic state after the different HIIT protocols, which can be considered an important adaptation. This result differed from that reported by Farzad et al. (2011) who did not find any significant difference in cortisol response to a 4-week HIIT program in wrestlers. In our study only the lower-body and the *uchi-komi* training groups increased the testosterone-cortisol ratio in response to training. A similar finding was reported by Farzad et al. (2011) in wrestlers submitted to high-intensity running training. Thus, as the lower-body and the *uchi-komi* training groups in the present study and the group investigated by Farzad et al. (2011), but not the upper-body training group in our study, involved large muscle mass, it is possible that this more anabolic state is only observed when the training stimulus result in a higher disturbance during each training session. Moreover, this more anabolic state might have contributed to the better performance after the training period as discussed above.

The present study found that short-term (4-week) low-volume (two blocks of 10 × 20 s/10 s all-out exercise) HIIT added to the regular judo training was able to induce increases in upper-body MAP when the upper-body training protocol was used, in upper-body power at OBLA for the lower-body training group, PP for the *uchi-komi* training group, MP for the lower-body training group in the fourth Wingate test in the lower-body intermittent test and in the testosterone-cortisol ratio in the lower-body intermittent test for the lower-body and *uchi-komi* training groups. No effects on any lower-body aerobic variable, muscle damage markers, body mass, and body fat percentage were found. The only negative effects found were the decreased MP in the lower-body intermittent test for the upper-body and the *uchi-komi* training groups.

It is important to observe that the different HIIT protocols result in upper-body aerobic and anaerobic performance improvement, while lower-body improvement ocurred only for anaerobic fitness measures. These results are probably due to the interaction between HIIT protocols and the high upper-body solicitation during judo training. Additionally, the *uchi-komi* protocol resulted in benefits for both upper- and lower-body parameters, probably due to the involvement of both segments during this training mode.

## Author contributions

Conceived and designed the experiments: EF, UJ. Performed the experiments: UJ, VP, BB. Analyzed the data: UJ, VP, JG, FL, EF. Wrote the paper: EF, UJ, VP, FL, JG, BB.

### Conflict of interest statement

The authors declare that the research was conducted in the absence of any commercial or financial relationships that could be construed as a potential conflict of interest.
